# Nicotine delivery and user reactions to Juul EU (20 mg/ml) compared with Juul US (59 mg/ml), cigarettes and other e-cigarette products

**DOI:** 10.1007/s00213-020-05734-2

**Published:** 2020-12-03

**Authors:** Anna Phillips-Waller, Dunja Przulj, Katie Myers Smith, Francesca Pesola, Peter Hajek

**Affiliations:** Health and Lifestyle Research Unit, Queen Mary University of London, 2 Stayner’s Road, London, E14AH UK

**Keywords:** E-cigarettes, Juul, Nicotine, Nicotine delivery, Pharmacokinetic, Tobacco

## Abstract

**Rationale:**

The degree to which the EU version of Juul with 20 mg/ml nicotine (Juul EU) delivers nicotine to users is likely to determine its treatment potential.

**Objectives:**

To compare the pharmacokinetic profile and user ratings of Juul EU, Juul US (59 mg/ml nicotine), cigarettes and other e-cigarette (EC) products.

**Methods:**

In a within-subjects crossover design, 18 vapers used, at separate sessions, their own brand cigarette (OBC), Juul US and Juul EU for 5 min ad libitum, after overnight abstinence. Seven of the participants also tested eight other EC previously. Blood samples were taken at baseline and 2, 4, 6, 8, 10 and 30 min after initiating product use. Products were rated on a range of characteristics.

**Results:**

Juul EU delivered less nicotine than OBC (t(13) = −4.64 *p* < .001) and than Juul US (t(13) = −6.40, *p* < .001): AUC_0 ≥ 30_ 77.3, 324.8 and 355.9, respectively. Maximum nicotine concentration (C_max_) was also much lower for Juul EU than Juul US (z = −3.59, *p* < .001): C_max_ 3.8 ng/ml vs 21.1 ng/ml, respectively. Juul EU was perceived to relieve urges to smoke less than Juul US (z = −2.29, *p* = .022) and to provide less nicotine (z = −2.57. *p* = 0.010). Juul EU delivered less nicotine than refillable EC (C_max_: t(6) = 3.02, *p* = 0.023; AUC_0 ≥ 30_: z = −2.20, *p* = 0.028) and also less than cig-a-like EC, though the difference did not reach significance (C_max_: t(6) = 2.49, *p* = 0.047; AUC_0 ≥ 30_: z = −1.99, *p* = 0.046). Subjective ratings of Juul EU and other EC products were similar.

**Conclusions:**

Juul EU delivers much less nicotine to users than Juul US, and also less than refillable EC products. It may thus have more limited potential to help smokers quit.

## Introduction

The US version of JUUL (‘Juul US’), a novel ‘pod’ based e-cigarette (EC), is currently the most popular EC in the USA (Kavuluru et al. 2019; King et al. 2018). Although it has several innovative features, the main reason for its popularity is likely to be its nicotine salt formulation, which is thought to make nicotine inhalation less irritating (Bowen and Xing 2015; Hajek et al. 2020), and thus allows the device to use a high nicotine concentration (59 mg/ml), a level that would be too irritating if used in traditional e-liquids.

We have recently demonstrated that with ad-lib use, nicotine delivery from Juul US is similar to that from cigarettes and that it is faster and higher than nicotine delivery from other ECs, despite participants taking fewer puffs from Juul US (Hajek et al. 2020). The product also received higher user ratings than other EC types. The implication of these findings is that Juul US may be more effective than other ECs in helping smokers to quit smoking but that it could also have greater addictive potential.

In the UK, the Tobacco Products Directive 2014/14/EU (TPD), introduced in May 2016, prohibits nicotine concentrations in e-liquid of over 20 mg/ml (Medicine and Healthcare products Regulatory Agency 2020). For this reason, Juul marketed in Europe (‘Juul EU’) uses nicotine concentration of maximum 20 mg/ml. This version delivers only about a third of nicotine into aerosol compared with Juul US (Talih et al. 2020). Delivery to users, however, is not known. Nicotine users can titrate nicotine intake by altering the frequency of puffing and depth of inhalation (Soar et al. 2019). It is thus possible that the lower delivery Juul EU could still provide nicotine levels that smokers seek.

We examined the pharmacokinetic (PK) profile of Juul EU, when used ad-lib by experienced vapers, and compared its parameters with those of Juul US, own-brand cigarettes, and other types of EC.

## Methods

### Study design

Within subjects, crossover design to determine the PK profile of Juul EU, and compare it with that of Juul US, cigarettes and other EC products, and to compare the different products in their effects on urge to smoke and product ratings.

### Participants

Eighteen healthy adults were recruited via social media and word of mouth. Participants were eligible if they were current daily vapers who smoked cigarettes at least occasionally and were willing to test a series of EC products and give blood samples.

### Procedures

Participants gave written informed consent and attended the laboratory after abstaining from smoking and vaping overnight. Abstinence from smoking was verified with a carbon monoxide (CO) reading of less than 10 ppm (ppm).

At the first session, participants smoked their own brand cigarette (OBC), which they provided. Juul US was tested at the next session, followed by Juul EU. Each session had at least 1 week wash out period before the next. Seven of the 18 participants also tested eight other ECs, as a part of an earlier study (Hajek et al. 2018; Hajek et al. 2017).

At each session an intravenous blood sampling line was placed in the participant’s arm, and a baseline sample of up to 5 ml was taken. Participants were then asked to smoke/vape (depending on which product was being tested) as much or as little as they wanted for 5 min. This is the time it usually takes to smoke a cigarette. Further samples of up to 5 ml each were then taken at 2, 4, 6, 8, 10 and 30 min after product initiation.

Blood samples were stored at −20C for up to 7 days before being transported to the laboratory for analysis.

The sessions took place between 7.30 am and 9.30 am depending on participant preferences and lasted approximately 1 h. Participants received £60 for each session.

## Measures

Demographic, smoking and vaping data were collected at the first session. Number of puffs were counted for each product tested. At each session, a baseline urge to smoke was rated (before product use) on a scale of 1 to 10, where 1 = no urge at all and 10 = extreme urge. Subsequent ratings were given 5, 10, 15 and 30 min after product initiation.

At all sessions the following questions were asked about the product used (rated on a scale of 1–10): ‘Did it relieve your urge to smoke?’ [not at all = 1, extremely well = 10]; ‘How quickly did any effect happen?’ [very slowly = 1, extremely fast = 10]; ‘How much nicotine do you think it delivered?’ [too little = 1, just right = 5, too much = 10]; ‘Did you like the taste?’ [not at all = 1, extremely = 10]; ‘Was it pleasant to use?’ [not at all = 1, extremely = 10]; ‘How likely would you be to recommend it to friends?’ [not at all = 1, extremely = 10] (the last question was not asked at the OBC session).

Nicotine in blood samples was analysed at ABS Laboratories Ltd., Bio Park (Welwyn Garden City, UK), using capillary column gas chromatography with detection by electron impact mass spectrometry and selected ion monitoring (Jacob III et al. 2011). The PK parameters calculated were maximum nicotine concentration (C_max_), time to the maximum (T_max_) and area under the curve (AUC_0 ≥ 30_), which is a measure of the total nicotine delivery over 30 min.

### Study products

Participants brought their OBC to test at the first session. Juul US (59 mg/ml nicotine content) and Juul EU (20 mg/ml nicotine content) were available with differently labelled tobacco flavours: Virginia Tobacco flavour in the case of Juul US and Golden Tobacco flavour in the case of Juul EU (JUUL Labs).

For the seven participants who previously tested eight traditional EC, tobacco flavour was used with nicotine contents as close to 20 mg/ml as possible, with the exception of Vuse (48 mg/ml), which was the highest nicotine concentration available at the time. The other products tested were five cig-a-likes: Blu (18 mg/ml), Vype (16.8 mg/ml), Puritane (20 mg/ml), E-lites (24 mg/ml) and Gamucci (16 mg/ml) and two refillables: KangerTech EVOD and InnokiniTaste MVP 2 (variable voltage), set to 4.8 V (range = 3.3–5.0 V). The refillable products were tested with the same 20 mg/ml tobacco flavour liquid (see Hajek et al. 2018; Hajek et al. 2017). All products were tested in the same order.

### Statistical analysis

PKSolver add-in for Excel version 2.0 (Zhang et al. 2010) was used to calculate C_max_, T_max_ and AUC_0 ≥ 30_, using a non-compartmental analysis and trapezoidal rule (Gabrielsson and Weiner 2001). The post product use blood samples were corrected for baseline nicotine levels.

Differences in post product use blood samples between Juul EU and Juul US and Juul EU and cigarette were analysed using *t* tests or a non-parametric equivalent when parametric assumptions were not met. We applied the Bonferroni correction for type I error.

Differences in product characteristics between Juul EU and Juul US, Juul EU and refillable EC and Juul EU and cig-a-like EC were analysed using *t* tests if parametric assumptions were met or Wilcoxon signed-rank test if not*.* We applied the Bonferroni correction for type I error where required.

Regarding changes in urges to smoke after product use (Juul EU and Juul US), we examined the main effects of Time and Product as well the Time*Product interaction on urges to smoke over the 30-min testing period using a mixed effect model, adjusting for baseline urge scores, with participants treated as the cluster. We also used random slopes and intercepts to account for repeated measures. The Wald test was used to assess the overall significance of Time, product and the Time*Product interaction.

We assessed the normality assumption using the Shapiro-Wilks test and through visual inspection of probability plots. For the mixed effect model, we visually assessed the assumption of homoscedasticity by plotting the standardized residuals against the fitted values. All analyses were performed with SPSS version 25 apart from the mixed-effect regression, which was run in Stata 16.

The project was approved by the QMUL Ethics of Research Committee on 3 April 2018 (QMERC2018/09).

## Results

Participant characteristics are show in Table 1. Seventeen (94%) participants were using refillable ECs.

**Table 1:** Participant characteristics (*N* = 18)

Age, median (IQR)	29.5 (25.8–41.0)
Male, N (%)	16 (88.9)
Higher Education, N (%)	11 (61.1)
Cigarettes smoked per day before starting EC use, median (IQR)	13.5 (5.8–20.0)
Fagerstrom Test for Cigarette Dependence (FTCD) before EC use, median (IQR)	4.0 (1.8–6.3)
Cigarettes smoked per day when joining the study, median (IQR)	0.7 (0.6–1.6)
Current nicotine strength liquid/cartridges used (mg/ml), median (IQR)	12.0 (5.3–17.3)
Millilitres of e-liquid used per day, median (IQR), *N* = 17	2.0 (1.2–4.2)
Number of months using EC daily, median (IQR)	12.0 (3.5–39.3)
Days EC used in last week, median (IQR)	7.0 (7.0–7.0)

### Comparison of Juul EU, Juul US and own brand cigarettes in nicotine delivery

**Fig. 1:**
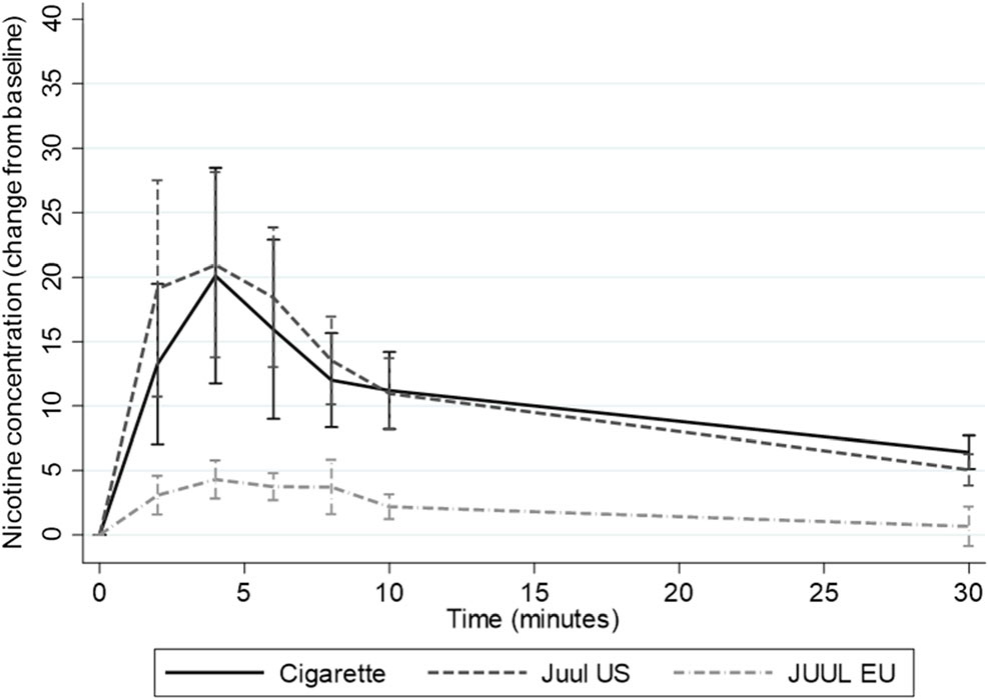
PK profiles of Juul EU, Juul US and own brand cigarette (*N* = 18) (mean scores with 95% CIs)

Juul EU delivered nicotine much more slowly and at lower levels than Juul US and cigarettes (see Fig. 1).

Table 2 shows the PK characteristics of Juul EU, Juul US and own brand cigarette, together with the number of puffs taken from each product during the 5 min of use. Maximum nicotine concentration (C_max)_ of Juul EU was significantly lower than both Juul US and own brand cigarette, as was total nicotine delivery over 30 min (AUC_0 ≥ 30)_. The difference between Juul EU and Juul US in time to maximum nicotine concentration did not reach statistical significance.

**Table 2:** Nicotine delivery and number of puffs taken from own brand cigarette, Juul EU and Juul US (*N* = 18)

Product	Median no. of puffs (IQR)	Median C_max_ (IQR)	Median T_max_^a^ (IQR)	Mean AUC_0 ≥ 30_^a^ (SD), *N* = 14 ^b^
Cigarette	13.5 (10.8–17.3)	12.9 (8.0–35.6)	5 (4–8)	324.8 (208.9)
Juul EU	15.5 (12.8–21.3)	3.8 (2.5–7.5)	6 (4–8)	77.3 (31.0)
Juul US	14.5 (10.0–21.5)	21.1 (9.9–36.3)	4 (2–6)	355.9 (173.7)
Difference between Juul EU and the other products^**c**^	Juul US: z = −0.76, *p* = 0.447 Cigarette: z = −1.83, *p* = 0.067	Juul US: z = −3.59, *p* < .001 Cigarette: z = −3.64, *p* < .001	Juul US: z = −1.82, *p* = 0.068 Cigarette: z = −0.52, *p* = 0.605	Juul US: t(13) = −6.40, *p *< .001 Cigarette: t(13) = −4.64, *p* < .001

^a^*Median T*_*max*_ *values and mean AUC*_*0 ≥ 30*_ *values that were used to compare products statistically differ slightly from values in* Fig. 1*estimated by PK Solver because the comparisons used means across individuals whereas PK Solver calculates means across time-points*

^b^*AUC*_*0 ≥ 30*_ *could not be calculated for four participants as the 30 min blood sample could not be collected*

^c^*Significance threshold set at 0.025 due to multiple testing*

Over all the test sessions, four participants had baseline nicotine levels of over 10 ng/ml at one or more sessions, indicating nicotine intake late at night or prior to the session. Excluding these participants in a sensitivity analysis did not change the results (Juul EU C_max_ versus Cigarette C_max_, Z = -3.17, *p* = 0.002; Juul US C_max_ versus Juul EU C_max_, Z = -3.30, *p* = 0.001; Cigarette AUC_0 ≥ 30_ versus Juul EU AUC_0 ≥ 30_, t(11) = 4.28, *p* = 0.001; Juul EU AUC_0 ≥ 30_ versus Juul US AUC_0 ≥ 30_, t(11) = −5.41, *p* < .001).

Table 3 compares participant reactions to Juul EU and Juul US. Juul EU relieved urges to smoke less than Juul US, and was perceived to deliver less nicotine.

**Table 3:** Participant ratings of Juul EU and Juul US (*N* = 18)

Product characteristic	Juul EU	Juul US	Difference
Did it relieve your urge to smoke (1–10), median (IQR)	8.5 (6.8–9.3)	9.0 (7.8–10.0)	z = −2.29, *p* = 0.022
How quickly did any effect happen? (1–10), median (IQR)	7.5 (6.0–8.0)	7.5 (7.0–9.0)	z = −1.27, *p* = 0.205
Subjective nicotine delivery (1 = too little, 5 = just right, 10 = too much), median (IQR)	5.0 (4.0–5.3)	5.5 (5.0–7.3)	z = −2.57, *p* = 0.010
Taste (1–10), mean (SD)	5.9 (2.2)	5.9 (2.6)	t(17) = 0.00, *p* = 1.00
Pleasantness (1–10), median (IQR)	8.0 (7.0–9.3)	7.0 (6.0–9.3)	z = −0.54, *p* = 0.592
Recommend (1–10), median (IQR)	7.0 (5.0–9.3)	7.0 (6.0–9.3)	z = −1.37, *p* = 0.170

**Fig. 2:**
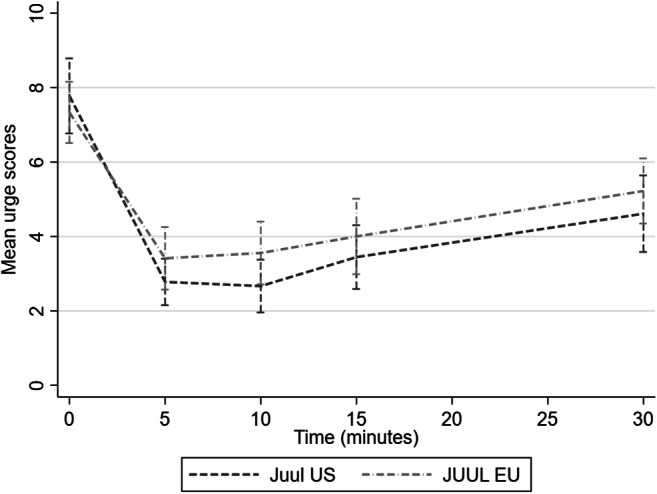
Effects of Juul EU and Juul US on urges to smoke over time

Figure 2 shows changes in urges to smoke over time after using Juul US and Juul EU. The mixed model analysis showed a main effect of time (Wald ×2(3) = 22.3, *p* < .001). Urges to smoke decreased 5 min after product use initiation, and subsequently increased, but not up to baseline levels. Ratings were lower when using Juul US (vs. Juul EU), but the difference did not reach significance for the main effect of product (Wald ×2(1) = 3.8, *p* = 0.052). There was no interaction effect between product and time (Wald ×2(3) = 0.6, *p* = 0.91).

### Comparison of Juul EU with other EC products

**Fig. 3:**
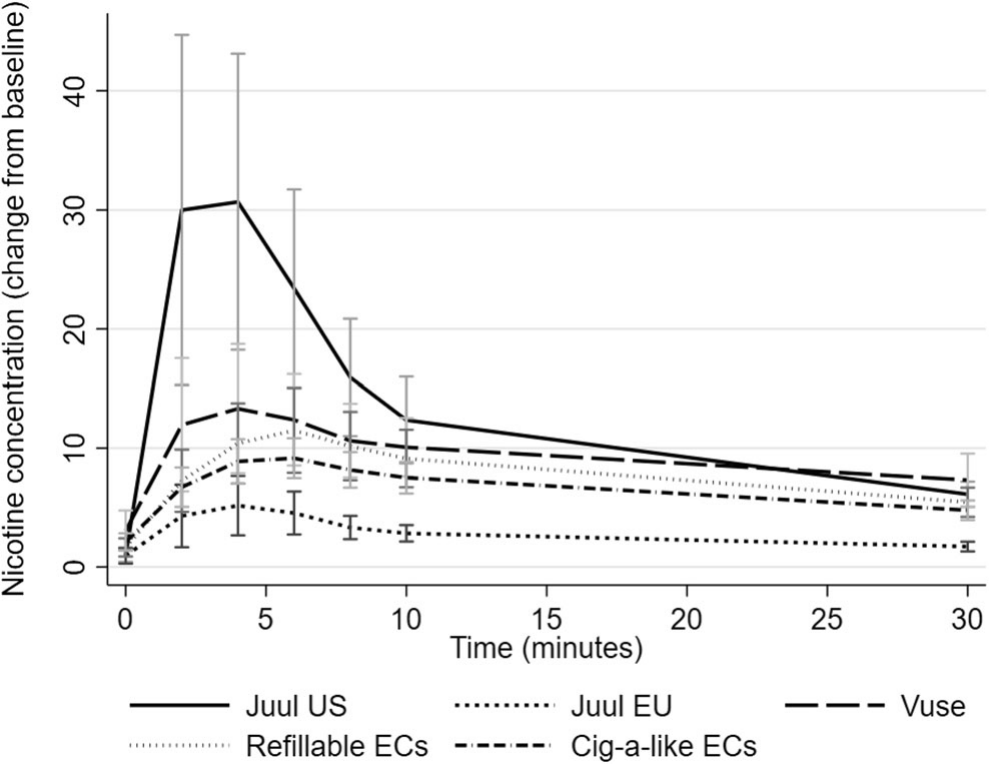
PK profiles of Juul EU, Juul US and eight traditional EC products (*N* = 7) (mean scores with 95% CIs)

Figure 3 shows the PK profiles of Juul EU, Juul US and the traditional EC products. The traditional ECs are grouped into cig-a-like and refillable. Vuse, which has a much higher e-liquid nicotine content (48 mg/ml), is shown separately.

Among these 7 participants, Juul EU had a significantly lower C_max_ than refillable EC and also lower than cig-a-like, though not significantly so. T_max_ did not differ significantly. There were no differences in number of puffs taken. Differences in total nicotine delivery approached but did not reach significance levels (see Table 4).

**Table 4:** Nicotine delivery and number of puffs taken from Juul EU, refillable and cig-a-like ECs (*N* = 7)

Product	Median no. of puffs (IQR)	Mean C_max_ (SD)	Mean T_max_^a^ (SD)	Median AUC_0 ≥ 30_^a^ (IQR), *N* = 6^b^
Juul EU	13.0 (11.0–16.0)	5.5 (3.4)	5.4 (2.5)	89.8 (71.4–106.9)
Refillable EC	14.5 (13.5–19.0)	10.5 (5.3)	10.0 (8.9)	203.2 (165.7–268.9)
Cig-a-like EC	15.8 (14.6–22.4)	8.0 (3.8)	8.1 (5.5)	136.6 (93.5–188.9)
Difference between Juul EU and the other products^**c**^	Refillable: z = −0.51, *p* = 0.611 Cig-a-like: z = −1.52, *p* = 0.128	Refillable: t(6) = 3.02, *p *= 0.023 Cig-a-like: t(6) = 2.49, *p* = 0.047	Refillable: t(6) = 1.30, *p* = 0.241 Cig-a-like: t(6) = 1.20, *p* = 0.275	Refillable: z = −2.20, *p* = 0.028 Cig-a-like: z = −1.99, *p* = 0.046

^a^*Median T*_*max*_ *values and mean AUC*_*0 ≥ 30*_ *values that were used to compare products statistically differ slightly from values in* Fig. 3*estimated by PK Solver because the comparisons used means across individuals whereas PK Solver calculates means across time-points*

^b^*AUC*_*0 ≥ 30*_ *could not be calculated for one participant as the 30 min blood sample could not be collected*

^c^*Significance threshold set at 0.025 due to multiple testing*

Over these product testing sessions, one participant had a baseline nicotine level of over 10 ng/ml. A sensitivity analysis was carried out with this participant excluded. There was no longer a significant difference in C_max_ between Juul EU and refillable EC: t(5) = 2.69, *p* = 0.043. Other results did not change.

Regarding product ratings, there was no significant difference between Juul EU and either cig-a-like or refillable EC on any measures (Table 5).

**Table 5:** Ratings of Juul EU and traditional ECs (*N* = 7)

Product characteristic	Refillable EC	Cig-a-like EC	Juul EU	Difference between Juul EU and other products^a^
Did it relieve your urge to smoke? (1–10), median, (IQR)	9.5 (7.5–10.0)	8.4 (6.2–9.2)	9.0 (7.0–10.0)	Refillable: z = −1.38, *p* = 0.168 Cig-a-like: z = −0.34, *p* = 0.734
How quickly did any effect happen? (1–10), mean (SD)	8.0 (1.1)	7.2 (1.3)	7.3 (1.5)	Refillable: t(6) = 1.22, *p* = 0.269 Cig-a-like: t(6) = −0.15, *p* = 0.886
Subjective nicotine delivery (1 = too little, 5 = just right, 10 = too much), median (IQR)	6.5 (6.0–7.5)	5.4 (5.0–6.6)	5.0 (4.0–5.0)	Refillable: z = −2.23, *p* = 0.026 Cig-a-like: z = −1.52, *p* = 0.128
Taste (1–10), median (IQR)	5.5 (4.0–6.0)	5.4 (4.8–6.4)	6.0 (5.0–7.0)	Refillable: z = −0.85, *p* = 0.395 Cig-a-like: z = −0.68, *p* = 0.498
Pleasantness (1–10), median (IQR)	5.5 (4.5–7.5)	6.0 (5.6–7.2)	7.0 (5.0–9.0)	Refillable: z = −0.42, *p* = 0.674 Cig-a-like: z = −0.51, *p* = 0.610
Would recommend to friends (1–10), mean (SD)	6.5 (1.7)	5.3 (2.0)	6.1 (3.3)	Refillable: t(6) = 0.24, *p* = 0.816 Cig-a-like: t(6) = −0.63, *p* = 0.551

^a^*Significance threshold set at 0.025 due to multiple testing*

## Discussion

When used ad lib over 5 min, the European version of Juul delivers much less nicotine to users than cigarettes and than the US Juul version. Juul EU’s C_max_ is also lower than that from refillable EC products used with the same nicotine concentration in e-liquid. Nicotine delivery from Juul EU was also marginally lower than that from cig-a-like products, which use similar battery power, although the difference was not significant. When used with a low nicotine concentration, the nicotine salt formulation does not seem to lead to improved nicotine delivery.

The difference in nicotine delivery between Juul EU and Juul US was reflected in participants’ subjective ratings of how much nicotine the products delivered (although the lower nicotine delivery from Juul EU was not perceived as a negative feature). This awareness of nicotine levels contrasts with our previous studies (Hajek et al. 2018; Hajek et al. 2017) where participants did not detect the nicotine content of EC well, not even the high nicotine content of Juul US, when used for the first time. We hypothesised that the nicotine salt formulation may have blunted sensory cues to Juul US’ high nicotine content (Hajek et al. 2020). With low nicotine delivery, the difference was noted.

Regarding sensory effects, the nicotine salt formulation is expected to reduce irritant effects of inhaled nicotine (Bowen and Xing 2015). We previously reported that Juul US was rated as more pleasant and more likely to be recommended to other smokers than other EC products (Hajek et al. 2020). In contrast, Juul EU did not differ in these ratings from other EC products. The benefit of the nicotine salt formulation on user experience may be seen only when high nicotine concentrations are used.

Corresponding with the lower nicotine delivery, Juul EU was also perceived to reduce urges to smoke less than Juul US. The differences in post product use ratings of urges to smoke trended in the same direction, but it did not reach significance.

Juul EU’s nicotine delivery was marginally lower than that from the first generation cig-a-like EC products, though on the reduced sample available for this comparison, the difference did not reach significance. Cig-a-like ECs are now rarely used, especially by established vapers (ASH 2019). Despite retaining the ease of use and the discrete appearance of Juul US, our results suggest that Juul EU is likely to be less appealing to smokers. Given the nicotine delivery profile and effects on urges to smoke, it may be less effective in helping smokers quit then Juul US, and probably also less than refillable EC products.

The findings support questions raised about the rationale of the nicotine content limit for e-cigarettes, stipulated in the EU TPD (Medicine and Healthcare products Regulatory Agency 2020). The regulation allows cigarettes to deliver the nicotine levels that smokers seek, but disallows much less risky competitive products to do so. The consequence of this could be that at least some smokers who could have switched to less dangerous alternatives with comparable nicotine delivery are unable to access them.

The study has several limitations. The sample comprised mostly of males. The sample size for the comparisons with other EC products was relatively small, although not unusually so for this type of study, and the differences in C_max_ and AUC_0 ≥ 30_ were significant, despite the subsample showing lower nicotine values than the full original sample (Hajek et al. 2017). Participants tested the two Juul products in the same order, and so order effects cannot be ruled out, although there was a gap of several months between the two sessions. The participants could see the products they were using. They were provided with no product information to avoid potential biases, but if they had any preconceived ideas about any of the products, this could have affected some of the results. The study tested product use ad libitum, to provide an approximation of real life use, rather than using a puffing schedule, that would provide information on nicotine intake per puff.

In conclusion, Juul EU does not deliver nicotine as effectively as either Juul US, or refillable EC, and may thus have more limited potential in helping smokers stop smoking.
